# Does physiological arousal lead to increased catastrophic misinterpretation? An experiment based on the concept of a fear memory

**DOI:** 10.1186/s40359-020-0384-y

**Published:** 2020-02-13

**Authors:** Barnabas Ohst, Brunna Tuschen-Caffier

**Affiliations:** Institut für Psychologie, Albert-Ludwigs-Universität Freiburg, Freiburg, Germany

**Keywords:** Catastrophic misinterpretation, Physiological arousal, Fear memory, Film manipulation

## Abstract

**Background:**

While there has been research on catastrophic misinterpretation of ambiguous situations and on the effects of the induction of physiological arousal, there has been no experimental research on the relationship between them. Based on the concept of a fear memory, we aimed to investigate if the induction of physiological arousal leads to catastrophic misinterpretations.

**Methods:**

Participants were shown either a suspenseful film clip to induce physiological arousal (EG, *n* = 43) or a calm film clip with no specific effect on arousal levels (CG, *n* = 40) before completing a measure of catastrophic misinterpretation (BSIQ-FR). To assess the specific predictive value of physiological arousal, measurements of other known predictors were included (BSI, BDI-II, ACQ, BSQ, STAI-T, ASI-3).

**Results:**

The film manipulation led to a significant increase in physiological arousal in the EG but not in the CG. The EG did not report more catastrophic misinterpretations than the CG – however, more participants in the EG reported at least one catastrophic misinterpretation. The increase in physiological arousal due to the film manipulation predicted catastrophic misinterpretation in the open response format in the EG, but not in the CG, even when controlling for other known predictors.

**Conclusions:**

Our study provides evidence that experimentally induced physiological arousal can predict catastrophic misinterpretation. The findings support the concept of a fear memory. With the BSIQ-FR, a German questionnaire measuring catastrophic misinterpretation was introduced. Further research on the relationship between physiological arousal and catastrophic misinterpretation with clinical samples is recommended.

## Background

According to cognitive models of panic, catastrophic misinterpretations of bodily sensations are assumed to lead to panic attacks (e.g., [1]). Moreover, physiological arousal associated with anxiety is often the source of panic-related bodily sensations according to the panic symptoms defined in DSM-5 (e.g., a pounding heart, sweating, or shortness of breath [2];). However, cognitive models of panic predict that any physiological arousal (e.g., arousal following physical exercise or the consumption of caffeine) can trigger catastrophic misinterpretation (e.g., [1]), not only arousal associated with anxiety.

### Catastrophic misinterpretation and the fear memory

This assumption is in line with the concept of a fear memory [3] which follows the idea that information about feared stimuli, physiological and behavioral responses, as well as information about the meaning of stimuli are stored in a network-like mental structure [4, 5]. Thus, it seems plausible that feared bodily sensations and other feared stimuli are stored in a fear memory along with associated catastrophic misinterpretations and their accompanying physiological and behavioral responses. The concept of a fear memory also assumes that this informational structure can be triggered by activating one of its components [6]. Furthermore, physiological arousal is seen as a necessary prerequisite for the activation of a fear memory [3].

In our experiment, we aim to trigger the fear memory by inducing physiological arousal via the presentation of a suspenseful film clip. We assume that, as a consequence, other elements of the fear memory (i.e., feared situations and catastrophic misinterpretations) will also be activated. A subsequently administered questionnaire aims at capturing the thus activated catastrophic misinterpretations by presenting ambiguous situations and asking participants to provide their interpretations.

### Research on catastrophic misinterpretation and the induction of physiological arousal

There has been research on catastrophic misinterpretation in patients with panic disorder, social anxiety disorder, generalized anxiety disorder, and healthy subjects (e.g., [7–10], for a review see [11]). There has also been extensive research on the effects of inducing physiological arousal via physical exercise (e.g., [12, 13]), or in the broader sense, via the ingestion of caffeine (e.g., [14–16]), or the inhalation of CO_2_ (e.g., [17–19]). However, to the best of our knowledge, there has been no experimental research investigating physiological arousal as an enhancer of catastrophic misinterpretation of feared stimuli. In most of the above studies, no cognitive measures were applied after the induction of bodily sensations [12–18]. In one of the few studies on this topic, catastrophic misinterpretation was assessed after the inhalation of CO_2_ by providing participants with a list of possible thoughts (e.g., “I am going to die”) that might have come into their mind after experiencing symptoms resulting from the inhalation of CO_2_ [19]. This approach, however, more captures catastrophic cognitions than catastrophic misinterpretations [20, 21].

### Rationale for the experiment

In the present study, we implemented an experimental setup based on the concept of a fear memory to measure catastrophic misinterpretation following the induction of physiological arousal. Since in past research, participants without a diagnosed anxiety disorder also reported catastrophic misinterpretations [7–10], we decided to recruit a non-clinical sample. To the best of our knowledge (see also [11]), the only established instrument to measure catastrophic misinterpretation is the Bodily Sensations Interpretation Questionnaire (BSIQ) in its various versions [8, 10, 22]. In the present experiment, we used an adapted German version of this instrument (i.e., the BSIQ-FR).

To activate the fear memory, we used a film manipulation to induce physiological arousal. Participants in the experimental group (EG) were shown a suspenseful film clip, whereas participants in the control group (CG) viewed a calm film clip. Film clips have proven to be a reliable method for eliciting emotions and concurrently physiological arousal in a laboratory setting [23]. We used a film clip to induce physiological arousal as compared to physical exercise, the ingestion of caffeine, or the inhalation of CO_2_ to avoid unwanted side effects such as chest pain [12], feelings of choking [14], or feeling faint [18]. Since physiological arousal is presumed to be sufficient to activate the components of a fear memory [3, 6], we wanted to limit the bodily effect of our experimental manipulation to avoid the possible effects of other and more uncomfortable bodily sensations. As a marker for physiological arousal we used skin conductance level (SCL), which is a typical indicator of autonomic nervous system activity (e.g., [24, 25]).

While our experiment aims to investigate if physiological arousal leads to catastrophic misinterpretations, other psychological characteristics have been found to be predictive of catastrophic misinterpretations (i.e., anxiety sensitivity [9], agoraphobic cognitions [26], and trait anxiety [26]). To be able to determine the specific predictive value of physiological arousal, we also included measurements of these characteristics.

We hypothesized that (1) the film manipulation would lead to a greater increase in physiological arousal in the EG as compared to the CG, (2) participants in the EG would report more catastrophic misinterpretations following the induction of physiological arousal than in the CG, and (3) the increase in physiological arousal would be a predictor of catastrophic misinterpretations in the EG, but not in the CG.

## Methods

### Participants

Participants were undergraduate students enrolled for a Bachelor’s degree in Psychology at the University of Freiburg. They were granted research credit for their participation in the experiment. Inclusion criteria were no diagnosed mental disorder (present or in the past), no history of panic attacks and good proficiency in German. Non-native speakers’ proficiency in German was evaluated in the pre-experimental talk. Additionally, their open responses were screened after participation. Seven participants were excluded from analysis, four due to technical problems (e.g., no recording of SCL data) and three due to insufficient proficiency in German. The final sample consisted of 83 participants (EG = 43, CG = 40). For sociodemographic data and basic clinical characteristics, see Table 1. Since the fear of bodily sensations (BSQ) is likely to affect the catastrophic misinterpretation of bodily sensations as measured by 11 out of 18 items of the BSIQ-FR, the BSQ score was included as covariate in all further analyses.

**Table 1:** Sociodemographic data and basic clinical characteristics

	Experimental Group (*n* = 43)	Control Group (*n* = 40)	*t-*test / *χ*^2^-test
Age	21.49 (3.62)	21.63 (3.26)	*t*(81) = *−*.18, *p* = .86
Gender	33 female (77%)	33 female (83%)	*χ*^2^(1) = .42, *p* = .52
BSI (GSI)	.58 (.44)	.43 (.30)	*t*(81) = 1.75, *p* = .09
BDI-II	7.84 (7.41)	6.68 (5.88)	*t*(81) = .79, *p* = .43
STAI-T	39.95 (7.41)	38.45 (9.18)	*t*(81) = .82, *p* = .41
ASI-3	17.05 (11.48)	14.93 (8.81)	*t*(81) = .94, *p* = .35
BSQ	2.06 (.69)	1.72 (.55)	*t*(81) = 2.42, *p* < .05, *d* = .53
ACQ	1.46 (.53)	1.37 (.27)	*t*(81) = 1.03, *p* = .31
STAI-S^a^	35.30 (8.20)	33.43 (6.44	*t*(81) = 1.15, *p* = .25

^a^ State anxiety before the experiment

### Instruments

#### Physiological measure

Electrodermal activity (EDA) was measured at 400 Hz using the Varioport-II system (Becker Meditec GmbH, Karlsruhe, Germany). To reflect electrodermal sympathetic activity [27], two 11-mm inner diameter Ag/AgCl electrodes were placed on the middle phalanx of the middle and ring fingers of the non-dominant hand. They were filled with an electrode paste (0.5% saline in a neutral lotion) formulated for measuring skin conductance and resistance (TD-246, Mansfield Research and Development LLC, St. Albans, Vermont, USA). As a parameter of EDA, skin conductance level (SCL) was used. Data inspection and artifact corrections were conducted offline using ANSLAB [28] with version R2014b of MATLAB (The MathWorks, Inc., Natick, Massachusetts, USA). The SCL graphs were scanned manually to identify artifacts using ANSLAB [28]. No artifacts were identified and thus no artifacts removed. No filters were applied to the data and the data was not down-sampled. For statistical analyses, SCL data was aggregated in 1 min segments with five segments for each data point of interest (i.e., presentation of pictures of landscapes as baseline, presentation of the film clip, and the beginning of the completion of the BSIQ-FR), resulting in 15 1 min segments. As variable for the increase in SCL, the difference between the last minute of the film clip (“post-film”) and the last minute of the baseline (“pre-film”) were used to make sure the pictures/film clips could unfold their effects.

#### Film clips

The film clip used in the EG was chosen from a database of 64 emotion-eliciting film clips [29] in a stepwise selection process. The database comprises film clips that aim at eliciting fear, anger, sadness, disgust, amusement, tenderness, as well as emotionally neutral scenes. Our aim was to find a film clip that induced high arousal of negative valence with as little co-elicitation of fear as possible. The induction of fear was minimized to ensure that potential effects were the result of the induction of physiological arousal and not an epiphenomenon of fear. An excerpt from “Seven” (USA, 1995) was selected, in which a police officer threatens a criminal with a gun, after the criminal has revealed that he has killed the police officer’s pregnant wife. At the end of the scene, it is left open whether the police officer shoots the criminal. The selected film clip has a length of 5:51 min, an arousal rating of 5.69 (8th rank in the database, maximum: 6.12, minimum: 1.63), an anger rating of .99 (9th rank, max: 2.19, min: − 1.65), a disgust rating of 1.70 (22nd rank, max: 4.07, min: − 1.70), a sadness rating of − 0.13 (23rd rank, max: 2.32, min − 1.47), and a fear rating of .47 (25th rank, max: 2.93, min: − 1.91). The ratings are discreteness coefficients: the mean score of the scale targeting one particular emotion (range: 1 to 7) minus the averaged mean scores of the scales targeting the other five emotions. A negative value indicates that the score of the targeted emotion is below the mean score of the other emotions.

For the CG, an excerpt from a garden documentary about mulching (“Querbeet”, Germany, 2016) was chosen. This film clip has a comparable length (5:50 min) and does not depict any objects with high potential for eliciting a phobic reaction in participants (e.g., spiders, snakes, heights). No ratings on its effects were available prior to our experiment.

#### Body sensations interpretation questionnaire-Freiburg (BSIQ-FR)

The BSIQ-FR is a modified German version of the BSIQ-M by Austin and Richards [8]. Both are adaptations of the BSIQ by Clark et al. [10], which is based on the Interpretation Questionnaire (IQ) by McNally and Foa [22]. Satisfactory test-retest reliability over 3 months was reported for the brief version of the BSIQ for patients with panic disorder for ranked responses (.73 for bodily sensations and .75 for external events [10]).

The BSIQ-M was first translated into German by the first author, then retranslated into English by a clinical expert whose first language was English. Discrepancies between the retranslated and the original version of the BSIQ-M were discussed and the German version adjusted accordingly. The German version of the BSIQ-M was then modified into the BSIQ-FR, as described below.

The BSIQ-FR includes all 18 items of the BSIQ-M. They fall into two categories: internal events (i.e., bodily sensations, *n* = 11) and external events (*n* = 7). External events include social events (e.g., being ignored by a shop assistant, *n* = 4) and general events (e.g., smelling smoke, *n* = 3). Each item consists of two parts: in the first part, participants are presented with an ambiguous situation (e.g., “You notice that your heart is beating quickly and pounding.”) and are asked to provide an explanation (“Why?”). In the second part, participants are presented with three potential explanations for the given situation (e.g., “Because you have been physically active.”) and are asked to rank them in the order in which they would be most likely to come to mind in the given situation.

In the IQ and the BSIQ, one of the explanations provided for items concerning bodily sensations is harm-related and two are benign, while in the BSIQ-M one benign option was replaced by an anxiety-related option. Since the meaning of anxiety-related responses is disputed [10, 30], we decided to replace the anxiety-related explanation with a benign explanation. Specifically, for the items 1, 6, 8, 9, and 13, we reinstated the benign explanation of the BSIQ and for the items 2, 4, 5, 11, 16, and 18, we created new benign explanations.

In the BSIQ-M, the prompt for the initial interpretation (“Why?”) is followed by the question “And then what might happen?” to probe if an initially anxiety-related response (e.g., “I’m having an anxiety attack.”) is only a precursor to an expected catastrophic outcome. However, in both previous studies using the BSIQ-M, anxiety-related initial interpretations were only followed by harm-related outcome responses in very few cases (435 and 316 anxiety-related initial interpretations were followed by 3 and 22 harm-related outcome responses in [7, 8], respectively). Therefore, we decided to omit this follow-up question and the subsequent question “If this outcome did happen, how bad an experience would it be for you?”

As in previous versions of the BSIQ, the open responses concerning bodily sensations are coded as harm-related (e.g., “I will have a heart attack.”), anxiety-related (e.g., “I will have a panic attack.”), or benign (e.g., “I did sports.”) and the open responses concerning external events are coded as harm-related (e.g., “The house is on fire.”) or benign (e.g., “Someone is smoking.”). Since no difference in the interpretation of bodily sensations and external events was expected in the present non-clinical sample, only one score including both item categories was calculated. Since for external events there is no anxiety-related code for open responses, only a harm-related score was calculated for open responses. For ranked responses, a score was computed based on the rank the harm-related explanation was given by participants (first rank = 3 points, second rank = 2 points, third rank = 1 point).

The BSIQ-FR was implemented as a computer-based questionnaire using the software EFS Survey (Questback GmbH, Cologne, Germany) in order to avoid missing values and to facilitate the scoring process. To ensure comparability with previous research using paper and pencil questionnaires, the layout of the input forms was closely matched with the layout of the BSIQ-M and the BSIQ.

#### Positive and negative affect schedule-modified (PANAS-M)

To control for anxiety-inducing effects of the film clip and to assess changes in attentiveness as self-report equivalent to physiological arousal, a modified version of the Positive and Negative Affect Schedule (PANAS, [31]; German version: [32]) was administered before and after the presentation of the film clip. The PANAS consists of 20 items concerning positive and negative emotional states that are rated on a 5-point Likert scale, ranging from 1 (“a little or not at all”) to 5 (“extremely”). The German version of the PANAS has shown good internal consistency with Cronbach’s *α* = .85 and *α* = .86 for the positive and negative affect items, respectively [32]. For the present study, a modified version of the PANAS comprising only six items was used. Its three negative affect items relating to anxiety and fear (“scared”, “afraid”, and “nervous”) were used as measure of anxiety and three positive affect items (“attentive”, “interested”, and “alert”) were used as measure of attentiveness.

#### Brief symptom inventory (BSI)

The Brief Symptom Inventory (BSI, [33]; German version: [34]) was included in the post-experimental set of measurements to assess overall mental stress, allowing us to control for differences between the EG and the CG. The BSI consists of 53 items across 9 dimensions concerning a variety of bodily, emotional and cognitive symptoms than can occur when people are mentally stressed. Each item is rated on a 5-point Likert scale, ranging from 0 (“not at all”) to 4 (“very strong”). The Global Severity Index (GSI; mean score of all responses) serves as an indicator for overall mental stress. The German version of the BSI has shown satisfactory internal consistency for the nine dimensions (*α* ≥ .70) and good internal consistency for the GSI (Cronbach’s *α* = .96) [35].

#### Beck depression inventory (BDI-II)

To assess depressive symptomatology which can also cause negative interpretations [36], the Beck Depression Inventory-II (BDI-II, [37]; German version: [38]) was included in the post-experimental set of measurements. The BDI-II consists of 21 items that assess the severity of depressive symptoms. Each item is rated from 0 to 3 according to severity. The German version of the BDI-II has shown good internal consistency (Cronbach’s *α* ≥ .89) in different clinical and non-clinical samples and satisfactory test-retest reliability (*r* = .78) in non-clinical samples [38].

#### Body sensations questionnaire (BSQ)

The Body Sensations Questionnaire (BSQ, [39]; German version: [40]) consists of 17 items concerning bodily sensations that can occur when people feel nervous or anxious. Participants are asked to rate how afraid they are of each bodily sensation on a 5-point Likert scale, ranging from 1 (“not at all”) to 5 (“extremely”). Since eleven of its items correspond to bodily panic symptoms, the BSQ can be interpreted as a measure of panic-specific anxiety sensitivity, which has been shown to be predictive of catastrophic misinterpretation [9] and was therefore included in the post-experimental set of measurements. The German version of the BSQ has shown good internal consistency (Cronbach’s *α* ≥ .80) in different clinical and non-clinical samples and satisfactory test-retest reliability (*r* ≥ .63) for patients with panic disorder or panic attacks [40].

#### Agoraphobic cognitions questionnaire (ACQ)

Agoraphobic cognitions have been shown to be predictive of catastrophic misinterpretations [26]. Therefore, the Agoraphobic Cognition Questionnaire (ACQ, [39]; German version: [40]) was included in the post-experimental set of measurements. The ACQ consists of 14 items concerning thoughts and ideas that can occur when people feel nervous or anxious. Each item is rated on a 5-point Likert scale, ranging from 1 (“never”) to 5 (“always”). The German version of the ACQ has shown satisfactory internal consistency (Cronbach’s *α* ≥ .74) in different clinical and non-clinical samples and satisfactory test-retest reliability (*r* ≥ .75) for patients with panic disorder or panic attacks [40].

#### State-trait anxiety inventory (STAI)

Since trait anxiety has been shown to be a predictor of catastrophic misinterpretations [26], the scale for trait anxiety (STAI-T) of the State-Trait Anxiety Inventory (STAI, [41]; German version: [42]) was included in the post-experimental set of measurements. The scale for state anxiety (STAI-S) was administered at the beginning of the experiment to control for the effect of different levels of state anxiety between participants on their responses to the BSIQ-FR. The two scales consist of 20 items that are rated on a 4-point Likert scale, ranging from 1 (“not at all”, “almost never”) to 4 (“very”, “almost always”). The German version of the STAI has shown good internal consistency of Cronbach’s *α* ≥ .90 and *α* ≥ .88 for the STAI-S and the STAI-T, respectively [42].

#### Anxiety sensitivity inventory (ASI-3)

Anxiety sensitivity has been shown to be predictive of catastrophic misinterpretations [9]. Therefore, the Anxiety Sensitivity Inventory-3 (ASI-3, [43]; German version: [44]) was included in the post-experimental set of measurements. The ASI consists of 18 items concerning fear of bodily and cognitive symptoms and social consequences of fear. Each item is rated on a 4-point Likert scale, ranging from 1 (“don’t agree at all”) to 4 (“agree completely”). The German version of the ASI-3 has shown good internal consistency (*α* ≥ .86) in different samples [44].

### Procedure

All experiments were conducted in a laboratory at the Department of Psychology, Clinical Psychology and Psychotherapy, University of Freiburg. To keep context variables constant, the window shutters were kept closed at all times, the light was turned on, and the thermostat was set to a fixed temperature (approximately 20 °C). All parts of the experiment were conducted with a desktop PC.

After getting informed consent and placing the electrodes, participants were presented with pictures of landscapes for 5 min to obtain an SCL baseline. Participants then completed the STAI-S and the PANAS-M to assess the momentary level of anxiety and attentiveness before the presentation of the film clip. To induce physiological arousal, participants in the EG were then shown a 5 min film clip from the thriller “Seven” (USA, 1995). Participants in the CG were shown a 5 min film clip from a garden documentary about mulching (“Querbeet”, 2016, Germany). Both film clips were presented in the German language over headphones. To assess changes in the level of anxiety and attentiveness due to the presentation of the film clip, participants then completed the PANAS-M again. The STAI-S was not administered again to ensure that the arousal-inducing effect of the film clip in the EG would carry over into the completion of the BSIQ-FR. Finally, participants completed the BSIQ-FR. The duration of the experiment varied between 60 and 90 min. SCL was measured throughout the experimental session. After completing the experiment, participants were offered the opportunity to ask questions.

To avoid an effect of the experiment on the completion of the additional questionnaires (BSI, BDI-II, ACQ, BSQ, STAI-T, ASI-3), participants were sent a link via e-mail to the questionnaires on the online platform EFS Survey (Questback GmbH, Cologne, Germany) 3 days after the experiment.

### Statistical analysis

To determine the effects of the film manipulation, two-way ANCOVAs with the factors Group (EG vs. CG) and Time (pre- vs. post-film) with repeated measures on the last factor and variables found to show significant differences between the groups as covariates were calculated for the anxiety and the attentiveness score of the PANAS-M. For arousal, a third level was introduced to the factor Time (pre-film vs. post-film vs. BSIQ), which captured the SCL 5 min into the completion of the BSIQ-FR. This is to determine whether increased arousal after the presentation of the film clip carried over into the completion of the BSIQ-FR. Additionally, the difference between anxiety scores pre- and post-film were entered as a further covariate to ensure that an increase in physiological arousal was not merely based on an increase in anxiety.

*T*-tests were used to compare the amount of harm-related open responses and the score for ranked responses between the groups and a *Chi*^*2*^-test was used to compare the number of participants with at least one catastrophic misinterpretation between the groups. Effect sizes are classified as small (d ≥ 0.2), medium (d ≥ 0.5), or large (d ≥ 0.8), in accordance with Cohen [45].

To determine potential predictors of catastrophic misinterpretation, multiple regressions with step-wise inclusion of variables were calculated for the amount of harm-related open responses and the score for ranked responses. Anxiety sensitivity (ASI-3), trait anxiety (STAI-T), state anxiety before the experimental intervention (STAI-S), fear of bodily sensations (BSQ), anxiety-related thoughts (ACQ), and the increase in anxiety (PANAS-M) and SCL (for both: difference between post- and pre-film scores) were entered as variables. The increase in anxiety was included to ensure that a potential predictive value of the increase in SCL is not merely based on an increase in anxiety.

The required sample size was calculated using G-Power [46, 47]. For all calculations, effect size was set to medium, alpha error to .05, and power to .8. For the analyses of the effects of the film manipulation (Hypothesis 1, ANCOVAs with repeated measures), the required total sample size was determined to be 128. To compare the amount of catastrophic misinterpretations between groups (Hypothesis 2, *t*-tests), the required total sample size was determined to be 126. To determine potential predictors of catastrophic misinterpretations for each group (Hypothesis 3, linear multiple regressions), the required sample size for each group was determined to be 103. Post-hoc power analyses will be presented in the Discussion section.

All statistical analyses were conducted with IBM SPSS Statistics for Windows, version 25.0 (IBM Corp., Armonk, New York, USA).

## Results

### Effects of the film manipulation

The film clip presented to participants before completing the BSIQ-FR was expected to induce arousal while eliciting as little anxiety as possible in the EG and to have no specific effect on arousal and/or mood in the CG. First, two-way ANCOVAs with the factors Group (EG vs. CG) and Time (pre- vs. post-film) with repeated measures on the last factor and the BSQ-score as a covariate were calculated for the anxiety and the attentiveness score of the PANAS-M.

**Fig. 1:**
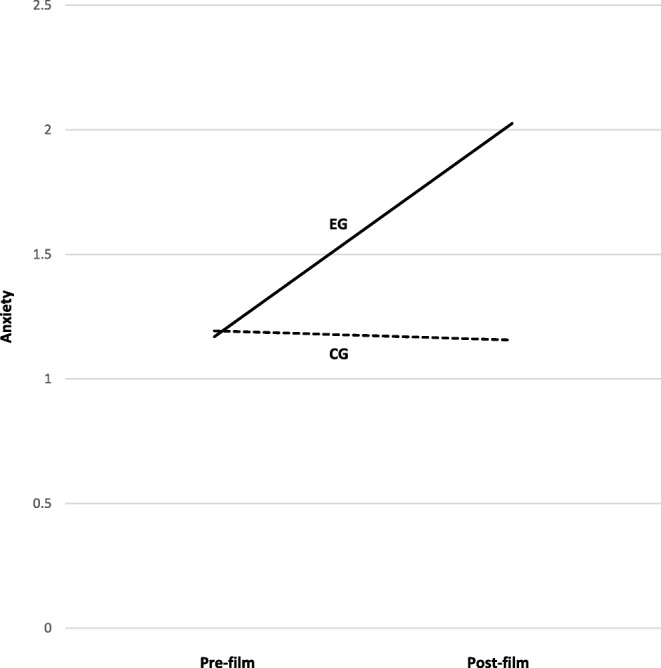
Increase in anxiety due to presentation of the film clip separated by group

For anxiety, a significant interaction of Group and Time was found, *F*(1, 80) = 45.19, *p* < .001, *η*^2^_p_ = .36, and a main effect for Group, *F*(1, 80) = 19.20, *p* < .001, *η*^2^_p_ = .19. The BSQ-score was a significant covariate, *F*(1, 80) = 8.06, *p* < .01, *η*^2^_p_ = .09, see Fig. 1. Post-hoc *t*-tests showed that before the presentation of the film clip, there was no significant difference between the groups (EG: *M* = 1.19, *SD* = .38, CG: *M* = 1.17, *SD* = .24, *p* = .70), while after the presentation the EG showed a significantly higher anxiety score (EG: *M* = 2.07, *SD* = .92, CG: *M* = 1.11, *SD* = .22, *p* < .001, *d* = 1.41). We found a small negative effect for anxiety in the CG (Pre-Film: *M* = 1.17, post-film: *M* = 1.11, *p* = .13, *d* = −.26) and a large effect in the EG (Pre-Film: *M* = 1.19, post-film: *M* = 2.07, *p* < .001, *d* = 1.25).

**Fig. 2:**
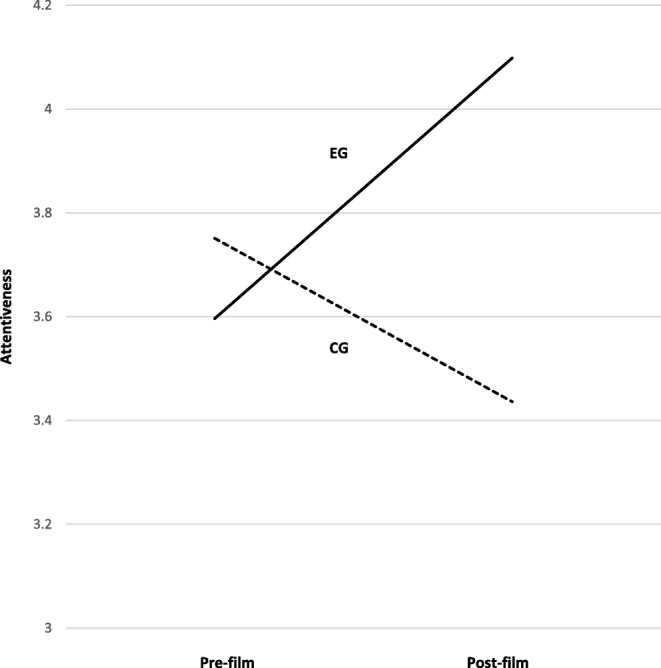
Increase in attentiveness due to presentation of the film clip separated by group

For attentiveness, a significant interaction of Group and Time could be found, *F*(1, 80) = 33.99, *p* < .001, *η*^2^_p_ = .30 but no significant main effects, see Fig. 2. Post-hoc *t*-tests showed that before the presentation of the film clip, there was no significant difference between the groups (EG: *M* = 3.58, *SD* = .56, CG: *M* = 3.77, *SD* = .61, *p* = .15), while after the presentation the EG showed a significantly higher attentiveness score (EG: *M* = 4.08, *SD* = .68, CG: *M* = 3.46, *SD* = .76, *p* < .001, *d* = .86). We found a small negative effect for attentiveness in the CG (Pre-Film: *M* = 3.77, post-film: *M* = 3.46, *p* < .01, *d* = −.45) a large effect in the EG (Pre-Film: *M* = 3.58, post-film: *M* = 4.08, *p* < .001, *d* = .80).

**Fig. 3:**
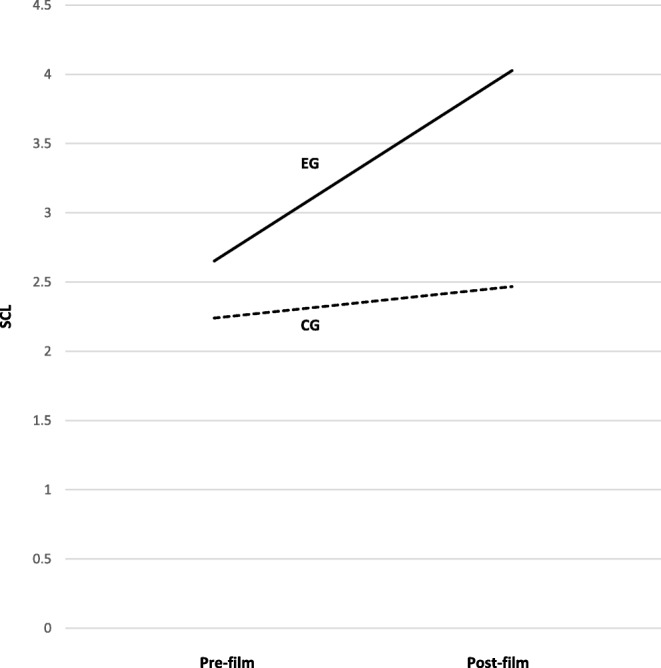
Increase in SCL due to presentation of the film clip separated by group

For arousal, a third level was introduced to the factor Time (pre-film vs. post-film vs. BSIQ), which captured the SCL 5 min into the completion of the BSIQ-FR. This is to determine whether increased arousal after the presentation of the film clip carried over into the completion of the BSIQ-FR. Additionally, the difference between anxiety scores pre- and post-film were entered as a further covariate to ensure that an increase in physiological arousal was not merely based on an increase in anxiety. In this analysis, a significant interaction of Group and Time could be found, *F*(1.77, 139) = 10.62, *p* < .001, *η*^2^_p_ = .12, while neither covariate was found to be significant, both *F*s < .8 and both *p*s > .37, see Fig. 3.

Post-hoc *t*-tests showed that before the presentation of the film clip, there was no significant difference between the groups (EG: *M* = 2.50, *SD* = 1.80, CG: *M* = 2.40, *SD* = 1.87, *p* = .80), while after the presentation the EG showed a significantly higher SCL (EG: *M* = 3.91, *SD* = 2.60, CG: *M* = 2.59, *SD* = 1.86, *p* < .01, *d* = .58). Five minutes into the completion of the BSIQ-FR, no significant difference was found (EG: *M* = 4.22, *SD* = 2.54, CG: *M* = 3.57, *SD* = 2.07, *p* = .20). We found no effect for SCL in the CG (Pre-Film: *M* = 2.40, post-film: *M* = 2.59, *p* < .05, *d* = .10) and a medium effect in the EG (Pre-Film: *M* = 2.50, post-film: *M* = 3.91, *p* < .001, *d* = .63).

### Catastrophic misinterpretation

Concerning the amount and ranking of catastrophic misinterpretations as measured by the BSIQ-FR, no significant differences were found between the EG and the CG for harm-related open responses and ranked responses, see Table 2. However, when comparing the number of participants with at least one catastrophic misinterpretation in the open response format, there was a significant difference between the conditions with more participants with at least one catastrophic misinterpretation in the EG (*n* = 37) than in the CG (*n* = 26), *χ*^2^(1) = 5.02, *p* < .05, *ϕ* = .25.

**Table 2:** Scores for harm-related open and ranked responses by experimental condition

	Experimental Group (*n* = 43)	Control Group (*n* = 40)	*t*-test
Open responses	.090 (.07)	.097 (.11)	*p* = .75
Ranked responses	1.40 (.27)	1.36 (.31)	*p* = .56

Note. Range for open responses: 0 to 1 (percentage of harm-related responses); range for ranked responses 1 to 3

### Predictors of catastrophic misinterpretation

To assess potential predictors of catastrophic misinterpretation, multiple regressions with step-wise inclusion of variables were calculated separately for harm-related open responses and the score for ranked responses. Anxiety sensitivity (ASI-3), trait anxiety (STAI-T), state anxiety before the experimental intervention (STAI-S), fear of bodily sensations (BSQ), anxiety-related thoughts (ACQ), and the increase in anxiety (PANAS-M) and SCL (for both: difference between post- and pre-film scores) were entered as variables. The increase in anxiety was included to ensure that a potential predictive value of the increase in SCL is not merely based on an increase in anxiety. For open responses, anxiety sensitivity and the increase in SCL were significant predictors of harm-related responses in the EG, while in the CG only trait anxiety reached significance, see Table 3. For ranked responses, anxiety sensitivity and agoraphobic cognitions were significant predictors of the harm-related score in the EG, while in the CG anxiety sensitivity and trait anxiety reached significance, see Table 4.

**Table 3:** Multiple regressions for harm-related open responses, separated by group

Experimental Group				Control Group			
	*b*	*SE b*	*β*		*b*	*SE b*	*β*
Step 1							
Constant	.027	.017		Constant	−.11	.07	
ASI-3	.004	.001	.58***	STAI-T	.005	.002	.44**
Step 2							
Constant	.004	.018					
ASI-3	.003	.001	.49***				
SCL	.022	.009	.32*				

Note. EG: *R*^2^ = .33 for Step 1; Δ*R*^2^ = .10 for Step 2 (*p*s < .05). CG: *R*^2^ = .19 for Step 1 (*p* < .01). *p < .05, **p < .01, ***p < .001

**Table 4:** Multiple regressions for ranked responses, separated by group

Experimental Group				Control Group			
	*b*	*SE b*	*β*		*b*	*SE b*	*β*
Step 1							
Constant	1.17	.06		Constant	1.09	.09	
ASI-3	.014	.003	.58***	ASI-3	.019	.005	.52***
Step 2							
Constant	.99	.10		Constant	.73	.18	
ASI-3	.008	.004	.36*	ASI-3	.013	.005	.37*
ACQ	.18	.08	.35*	STAI-T	.012	.005	.34*

Note. EG: *R*^2^ = .33 for Step 1; Δ*R*^2^ = .07 for Step 2 (*p*s < .05). CG: *R*^2^ = .27 for Step 1; Δ*R*^2^ = .09 for Step 2 (*p*s < .05). *p < .05, **p < .01, ***p < .001

## Discussion

Following the concept of a fear memory [3], our experiment aimed to investigate the relationship between physiological arousal and catastrophic misinterpretations. Specifically, we tested whether the induction of physiological arousal via a suspenseful film clip would enhance catastrophic misinterpretation.

As hypothesized, the film manipulation led to a greater increase in physiological arousal in the EG than in the CG, even when controlling for the concurrent increase in anxiety. The extent of the induction of arousal due to the film clip in the EG might have been limited by the non-clinical sample without pronounced anxiety (trait anxiety: *MW* = 39.23, *SD* = 8.28 for the STAI-T; non-clinical norm sample: *N* = 1141, *MW* = 35.03, *SD* = 8.36 [48]. If the increase in physiological arousal in the EG due to the film clip carried over into the subsequent completion of the BSIQ-FR cannot be conclusively determined, since participants typing in the responses could have increased SCL as well, as the electrodes were placed on the fingers. The increase in SCL in the CG in the first 5 min of completing the BSIQ-FR supports this assumption.

In line with previous research [7–10], participants in both the EG and the CG reported catastrophic misinterpretations. However, contrary to our hypothesis, participants in the EG did not report more catastrophic misinterpretations or ranked them higher than participants in the CG. Again, this finding might be a consequence of the non-clinical sample without a pronounced fear memory. Therefore, it is noteworthy that significantly more participants reported at least one catastrophic misinterpretation in the EG than in the CG. This result is an indicator that our experimental setup is able to activate catastrophic misinterpretations via the induction of physiological arousal.

In accordance with our third hypothesis, the increase in physiological arousal was a significant predictor of catastrophic misinterpretations in the open response format in the EG, but not in the CG. The increase in physiological arousal was the only significant predictor besides anxiety sensitivity in the EG and its inclusion led to 10% more explained variance. Anxiety sensitivity as a predictor is in line with previous findings [9]. For ranked responses, however, the increase in physiological arousal was not found to be a significant predictor for the ranking of harm-related response options for either the EG or the CG. The finding that physiological arousal is predictive for open but not for ranked responses is in line with an assumption by Harvey et al. [49]: They suggested that the harm-related response options in the ranking task activate threat-related cognitive schemata. Therefore, in the ranking task, the questionnaire itself might already sufficiently activate the fear memory and our experimental manipulation might not have added an incremental contribution to its activation. In the open response format, on the other hand, where no activation of threat-related cognitive schemata via the questionnaire occurs, our induction of physiological arousal might have been responsible for the activation of the fear memory, resulting in its contribution to the prediction of catastrophic misinterpretations.

It is noteworthy that we did not directly assess the activation of the fear memory. Rather, we hypothesized that the induction of arousal (operationalized as the increase in SCL) would activate the fear memory and thus enhance catastrophic misinterpretation (as measured by the BSIQ-FR). Therefore, our conclusions concerning the activation of the fear memory are indirect and based on the differences in catastrophic misinterpretation between EG and CG.

A limitation of our experiment is the potential influence of the negative valence of the film clip we used in the EG. We tried to control for the influence of anxiety induced by the film clip. However, the film clip may have evoked other negative emotions (e.g., anger) as well. A further limitation of our experiment is the sample size. A priori calculations of the required sample size to detect medium effects varied between 103 (Hypothesis 3) and 128 (Hypothesis 1). With 83 participants, the size of our final sample was considerably smaller, decreasing the statistical power of analyses.

Since the present experiment dealt with anxiety-related constructs, it is noteworthy that the prevalence of anxiety disorders is gender-dependent with a much higher prevalence amongst women than amongst men [50]. Therefore, results may vary depending on the composition of the sample. To avoid a bias, we made sure that there was a comparable percentage of female participants in both groups.

## Conclusion

The present study provides evidence that experimentally induced physiological arousal can predict catastrophic misinterpretation. The negative valence of the used stimuli, however, is likely to have played a role as well. Therefore, a replication with stimuli with a positive valence could help to clarify the role of physiological arousal. Nonetheless, the findings support the concept of a fear memory [3]. Additionally, with the BSIQ-FR, we introduced a German questionnaire measuring catastrophic misinterpretation. The findings of the present study were limited by the low trait anxiety of the non-clinical sample. Further research is recommended using a similar experimental approach with clinical samples that can be expected to have a more pronounced fear memory.

## Data Availability

The datasets used and/or analyzed during the current study are available from the corresponding author on reasonable request.

## Abbreviations

ACQ: Agoraphobic Cognitions Questionnaire
ASI-3: Anxiety Sensitivity Index
BBSIQ: Brief Bodily Sensations Interpretation Questionnaire
BDI-II: Beck Depression Inventory
BSI: Brief Symptom Inventory
BSIQ: Bodily Sensations Interpretation Questionnaire
BSIQ-FR: Bodily Sensations Interpretation Questionnaire-Freiburg
BSIQ-M: Bodily Sensations Interpretation Questionnaire-Modified
BSQ: Body Sensations Questionnaire
CG: Control group
EDA: Electrodermal activity
EG: Experimental group
GSI: Global Severity Index
IQ: Interpretation Questionnaire
PANAS: Positive and Negative Affect Schedule
PANAS-M: Positive and Negative Affect Schedule-Modified
SCL: Skin conductance level
STAI: State-Trait Anxiety Inventory
STAI-S: State-Trait Anxiety Inventory-State
STAI-T: State-Trait Anxiety Inventory-Trait
